# Eligibility and Safety of Triple Therapy for Hepatitis C: Lessons Learned from the First Experience in a Real World Setting

**DOI:** 10.1371/journal.pone.0055285

**Published:** 2013-02-01

**Authors:** Benjamin Maasoumy, Kerstin Port, Antoaneta Angelova Markova, Beatriz Calle Serrano, Magdalena Rogalska-Taranta, Lisa Sollik, Carola Mix, Janina Kirschner, Michael P. Manns, Heiner Wedemeyer, Markus Cornberg

**Affiliations:** Department of Gastroenterology, Hepatology and Endocrinology, Hannover Medical School, Hannover, Germany; Saint Louis University, United States of America

## Abstract

**Background:**

HCV protease inhibitors (PIs) boceprevir and telaprevir in combination with PEG-Interferon alfa and Ribavirin (P/R) is the new standard of care in the treatment of chronic HCV genotype 1 (GT1) infection. However, not every HCV GT1 infected patient is eligible for P/R/PI therapy. Furthermore phase III studies did not necessarily reflect real world as patients with advanced liver disease or comorbidities were underrepresented. The aim of our study was to analyze the eligibility and safety of P/R/PI treatment in a real world setting of a tertiary referral center.

**Methods:**

All consecutive HCV GT1 infected patients who were referred to our hepatitis treatment unit between June and November 2011 were included. Patients were evaluated for P/R/PI according to their individual risk/benefit ratio based on 4 factors: Treatment-associated safety concerns, chance for SVR, treatment urgency and nonmedical patient related reasons. On treatment data were analyzed until week 12.

**Results:**

208 patients were included (F3/F4 64%, mean platelet count 169/nl, 40% treatment-naïve). Treatment was not initiated in 103 patients most frequently due to safety concerns. 19 patients were treated in phase II/III trials or by local centers and a triple therapy concept was initiated at our unit in 86 patients. Hospitalization was required in 16 patients; one patient died due to a gastrointestinal infection possibly related to treatment. A platelet count of <110/nl was associated with hospitalization as well as treatment failure. Overall, 128 patients were either not eligible for therapy or experienced a treatment failure at week 12.

**Conclusions:**

P/R/PI therapies are complex, time-consuming and sometimes dangerous in a real world setting, especially in patients with advanced liver disease. A careful patient selection plays a crucial role to improve safety of PI based therapies. A significant number of patients are not eligible for P/R/PI, emphasizing the need for alternative therapeutic options.

## Introduction

Hepatitis C virus (HCV) infection remains a global health burden with approximately 160 million chronically infected individuals worldwide [1] including 8–11 million patients in Europe [2]. Chronic HCV infection is a major cause of liver cirrhosis and hepatocellular carcinoma [3], [4]. An effective antiviral treatment with sustained virological response (SVR) is associated with a significant improvement of the overall clinical outcome in particular at more advanced stages of the disease with severe liver fibrosis [5].

Combination therapy of pegylated interferon alfa and ribavirin (P/R) has been the standard of care since more than 10 years [6]. Recently the approval of the protease inhibitors (PI) boceprevir (BOC) and telaprevir (TLV) as first generation of new direct acting antivirals (DAA) has been a milestone in the therapy of chronic HCV genotype 1 infection. In phase III studies 67–75% of the therapy-naïve patients achieved SVR after a triple therapy consisting of P/R and PI [7], [8]. Even higher SVR rates of up to 80% were observed in those, who experienced a relapse after a previous therapy with P/R [9], [10]. In addition, the overall safety profile appeared to be moderate in these trials [7]–[10]. Despite these encouraging results there still remain some challenges ahead. SVR rates with PI-based triple therapies were much lower in patients with a previous null-response to P/R, especially in those individuals who also had advanced liver fibrosis and cirrhosis [11]. Furthermore, phase III trials do not necessarily reflect real world setting since the study population was highly selective. For example, patients with liver cirrhosis were underrepresented in these studies and those with advanced cirrhosis, low platelets or with additional risk factors like higher age or comorbidities were entirely excluded. Preliminary week 16 results of the French early access program (CUPIC) investigating the new triple therapy only in those with advanced liver fibrosis revealed a totally different safety profile with alarming rates of severe adverse effects (SAE) of up to 49% and a mortality rate of up to 2% [12]. In addition, it has to be considered that a certain part of the infected population is not eligible for the new therapies at the first place. Since current therapy concepts are still based on interferon alfa, several contraindications may prevent antiviral therapy. Various DAAs are currently in preclinical and clinical development and encouraging results have been published recently suggesting the introduction of interferon-free regimens in the near future [13]. Thus, it might well be a preferable alternative to wait for more efficient and safer treatment options in patients with only mild liver disease. In addition, limited resources may prevent treatment of all eligible patients.

The aim of our study was to analyze the eligibility and safety of new triple therapy concepts for the treatment of chronic HCV genotype 1 infection in a real world setting of a German tertiary referral center.

## Patients and Methods

### Patient Selection

**Figure 1:**
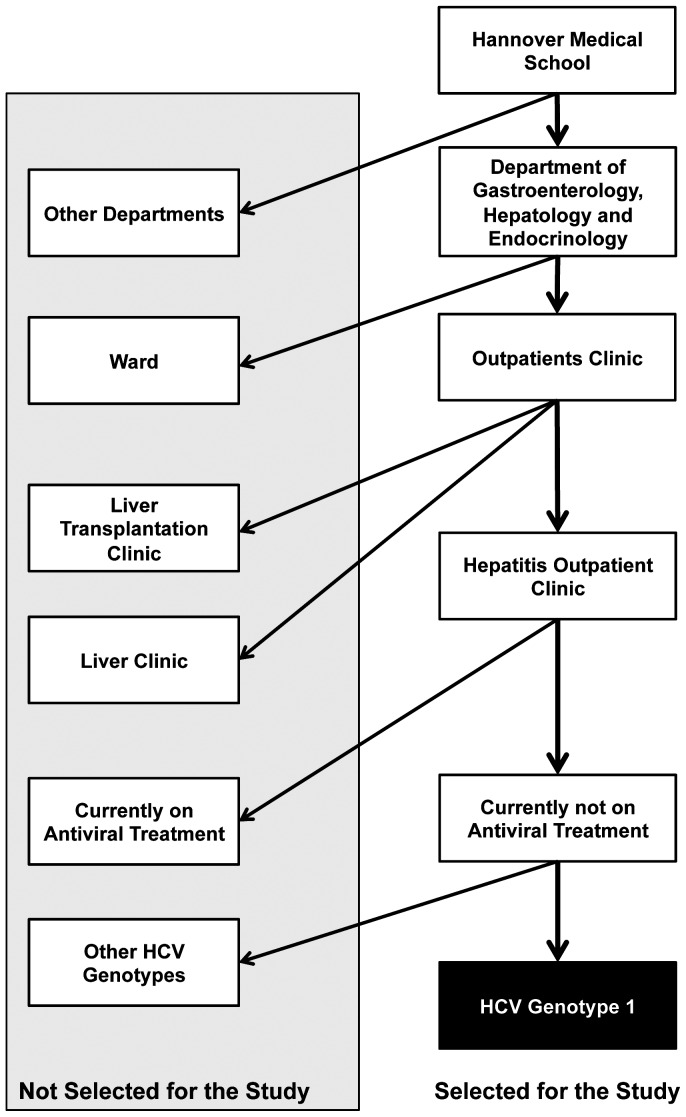
Selection of the study cohort.

All consecutive patients with chronic HCV genotype 1 infection who were referred to our hepatitis outpatient clinic between June 1^st^ and November 30^th^ 2011 were included. Excluded were patients with antiviral treatment at the time of their initial presentation during this time period. All patients were evaluated for a triple therapy concept. Figure 1 gives a schematic overview of the selection algorithm of our study. We recorded all reasons that influenced whether treatment was initiated or not until May 31^st^ 2012. Patient data were analyzed anonymously.

This study has been conducted according to the principles expressed in the Declaration of Helsinki. The ethical committee of Hannover Medical School approved this research project and waived the need for written informed consent because of the anonymous evaluation of patient data from patient records. For routinely assessment of IL28B genotype written informed consent was obtained.

### Assessment of Baseline Parameters

Routine laboratory parameters like hemoglobin level, platelet counts, ALT, AST and INR were measured by standard procedures. HCV RNA levels were detected using Roche COBAS TaqMan, Version 1. Extraction of the RNA was done automatically by COBAS AmpliPrep (Roche) according to the manufactures instructions. In those patients who gave written informed consent, assessment of the IL28B genotype (rs12979860) was performed as described previously using the Light Mix Kit rs12979860 TIB MOLBIOL [14]. The stage of liver fibrosis is described according to the METAVIR-Score. The majority of patients were classified using transient elastography/Fibroscan (84%). For classification the following cut off values were used: F0/F1:<7.1 kPa; F1/F2: ≥ 7.1 kPa; F2 ≥ 8.7 kPa; F3: ≥ 9.5 kPa; F3/F4: ≥ 12.5 kPa; Definite cirrhosis: ≥ 14.5 kPa [15], [16]. In the remaining cases the stage of fibrosis was determined based on a liver biopsy or obvious clinical parameters indicating liver cirrhosis.

### Triple Therapy Concepts

Different triple therapy concepts were considered. Some patients were treated with an individualized lead-in phase followed by the treatment protocol according to the approved label. These individualized concepts were planned for cases of uncertain treatment tolerability or low chances for SVR. In a few patients individualized lead-in phases included episodes with RBV mono-therapy prior to the standard BOC or TLV treatment protocol i.e. in cases of uncertain RBV tolerance or in some patients with thrombocytopenia with the purpose to increase or stabilize platelet count. Davis et al. have shown that IFN-induced decrease of platelet count was less pronounced if RBV is co-administered [17]. In addition we also used a P/R lead-in prior to the TLV label regimen with or without previous RBV mono-therapy. For patients who were started on a triple therapy concept at our hepatitis outpatient clinic until May 31^st^ 2012 safety and efficacy data were analyzed until treatment week 12 of the standard treatment regimen according to label. In those patients treated with an individualized lead-in concept week 0 of therapy was defined as the start of the approved standard treatment (4 weeks P/R, 24–44 weeks P/R/BOC or 12 weeks P/R/TLV).

### Definition of Treatment Failure

Treatment failure was defined as either virological failure or a permanent discontinuation of all antiviral medication due treatment intolerance i.e. in cases of AEs. Virological failure was defined along with the futility rules according to the respective labels: A) HCV RNA level ≥ 1,000 IU/ml at week 4 and/or week 12 of triple therapy including TLV. B) HCV RNA ≥100 IU/ml at treatment week 12 of a BOC protocol. C) Virological breakthrough was defined as an increase of HCV RNA level of>1 log.

In addition, patients with liver cirrhosis and previous null-response to P/R were also classified as virological failure, if they achieved<1 log decline of HCV RNA after a four-week lead-in phase with P/R. According to previously published studies chances for SVR have to be estimated as very low in these cases [18]. Thus we decided not to continue treatment due to an inadequate risk/benefit ratio.

### Statistical Analysis

All data are either presented as absolute numbers or as mean ± SD unless otherwise stated. Continuous data we analyzed with t-test and categorical data with χ2 tests.

## Results

### Patients and Evaluation Process

Baseline characteristics of the study cohort are shown in Table 1. 55% of the patients were male. Mean age was 52.9 years. The majority of patients were infected with HCV genotype 1b (62%) and the predominant IL28B genotype was CT (44%), whereas IL28B CC was present in only 18% of the individuals. Only 40% of the patients were treatment-naïve. Platelet counts below 150/nl were detected in 84 (40%) patients and 35 patients (17%) had platelets of<90/nl. The mean hemoglobin concentration was 14.3 g/dl including 21 individuals with hemoglobin levels<13 g/dl (men) or 12 g/dl (women). Baseline serum ALT levels were elevated in the majority of patients but only 15 patients had ALT levels of more than five times the upper limit of normal (ULN). Advanced liver fibrosis (F3/F4 according to METAVIR) was present in 133 (64%) individuals including 88 (42%) patients with definite cirrhosis. Only nine patients (4.3%) had a Child-Pugh Score of B indicating pre-selection of patients referred for antiviral therapy to our hepatitis treatment unit. Patients with decompensated cirrhosis are being referred to our liver transplant outpatient clinic.

**Table 1:** Baseline characteristics of the study cohort.

		All Patients (A)	Patients selected for Antiviral Treatment (B)	Patients Treated with a Triple Therapy Concept (C)	Not Treated (D)	p-value C vs. D
Patient number		208	105	86	103	
Scheduled for						
	TLV			61 (71%)		
	BOC			25 (29%)		
Mean Age ± SD (years)		52.9 ± 12	52.6 ± 10.1	53.5 ± 10.4	53.1 ± 13.7	0.41
Gender						
	Male (m)	115 (55%)	65 (62%)	55 (64%)	50 (49%)	0.16
	Female (f)	93 (45%)	40 (38%)	31 (36%)	53 (51%)	0.11
HCV genotype						
	1a	75 (36%)	34 (32%)	26 (30%)	41 (40%)	0.27
	1b	128 (62%)	69 (66%)	58 (67%)	59 (57%)	0.38
	Mixed/n.d.	5 (2.4%)	2 (1.9%)	2 (2.3%)	3 (3%)	0.80
IL28B genotype						
	CC	38 (18%)	12 (11%)	10 (12%)	26 (25%)	< 0.01
	CT	92 (44%)	55 (52%)	52 (60%)	37 (36%)	0.11
	TT	29 (14%)	17 (16%)	13 (15%)	12 (12%)	0.84
	N/A	49 (24%)	21 (20%)	11 (13%)	28 (27%)	
Treatment experienced						
	Yes	124 (60%)	70 (67%)	63 (73%)	54 (52%)	< 0.05
	No	84 (40%)	35 (33%)	23 (27%)	49 (48%)	0.07
Platelet counts (/nl)						
	*Mean ± SD*	169 ± 77.6	166 ± 69	158 ± 68.7	172 ± 85.4	0.11
	> 149	121 (58%)	60 (57%)	46 (53%)	61 (59%)	0.61
	90–149	49 (24%)	29 (28%)	25 (29%)	20 (19%)	0.17
	< 90	35 (17%)	14 (13%)	14 (16%)	21 (20%)	0.52
	N/A	3 (1.4%)	2 (1.9%)	1 (1.2%)	1 (1%)	
Hemoglobin (g/dl)						
	*Mean ± SD*	14.3 ± 1.77	14.5 ± 1.64	14.6 ± 1.61	14.2 ± 1.87	0.05
	<13 (m), <12 (f)	21 (10%)	6 (5.7%)	5 (5.8%)	15 (15%)	0.07
ALT						
	*Mean ± SD*	95.5 ± 81.6	102 ± 82.2	102 ± 76.4	88.9 ± 80.4	0.13
	< ULN	46 (22%)	16 (15%)	13 (15%)	30 (29%)	< 0.05
	1–2×ULN	82 (39%)	45 (43%)	36 (42%)	37 (36%)	0.51
	2–5×ULN	64 (31%)	35 (33%)	31 (36%)	29 (28%)	0.34
	> 5×ULN	15 (7.2%)	8 (7.6%)	6 (7%)	7 (6.8%)	0.96
	N/A	1 (0.5%)	1 (1%)	0 (0%)	0 (0%)	
Fibrosis Stage						
	F0–F2	72 (35%)	22 (21%)	11 (13%)	50 (49%)	< 0.0001
	F3/F4	133 (64%)	82 (78%)	74 (86%)	51 (50%)	< 0.01
	N/A	3 (1.4%)	1 (1%)	1 (1.2%)	2 (1.9%)	
De Ritis ratio						
	≤ 1	131 (63%)	69 (66%)	58 (67%)	62 (60%)	0.53
	> 1	76 (37%)	35 (33%)	28 (33%)	41 (40%)	0.41
	N/A	1 (0.5%)	1 (1%)	0 (0%)	0 (0%)	
Child-Pugh Score						
	5	167 (80%)	83 (79%)	67 (78%)	84 (82%)	0.78
	6	18 (8.7%)	10 (9.5%)	9 (10%)	8 (7.8%)	0.54
	> 6	9 (4.3%)	3 (2.9%)	2 (2.3%)	6 (5.8%)	0.24
	N/A	14 (6.7%)	9 (8.6%)	8 (9.3%)	5 (4.9%)	

TLV: telaprevir; BOC: boceprevir; N/A: not available; n.d.: not differentiated; SD: standard deviation.

Of the 208 patients, eleven were included into clinical phase II or III trials and were therefore not further considered for this analysis. Treatment was not initiated in 103 patients. The remaining 94 patients were considered for a triple therapy concept, of which eight preferred treatment at other centers. Thus, treatment was started in 86 patients at our hepatitis outpatient clinic. Patients who received antiviral therapy at our center were more likely to be male, to be infected with HCV genotype 1b, to have higher ALT levels and to show a more advanced stage of liver fibrosis than patients who were not treated. Treated patients were more often patients with previous treatment failure, which explains the lower prevalence of IL28B CC in the treated population (Table 1).

### Factors that Influenced the Decision not to Start P/R/PI

Four key factors were considered to calculate the risk/benefit ratio during evaluation process: (i) Therapy-associated safety concerns, (ii) chances for SVR, (iii) treatment urgency and (iv) nonmedical patient related reasons. Sometimes one of these factors completely dominated final decision i.e. in some patients with obvious interferon intolerance. Still, in many patients two or more of these factors significantly influenced the final decision indicating the complexity of the evaluation process.

- In 66 (64%) patients risk of SAEs during P/R/PI treatment was considerable. This was majorly related to comorbidities affecting 48 (47%) patients, most frequently the risk of exacerbation of an autoimmune reaction during treatment with interferon alfa since 18 patients were either recipients of organ transplants or had a history of an autoimmune disease. Severe psychological instability and disorders like severe depression i.e. with a history of a suicidal attempt or psychosis were relevant in 15 cases. Cardiovascular diseases i.e. a history of heart attacks, bypass or present congestive heart failure were important in eight and a low level of hemoglobin in seven individuals. Other comorbidities as impaired renal function, thyroidal dysfunction or severe diabetes were only relevant in a few patients. Besides comorbidities liver related morbidity played an essential role as well. Overall, in 10 patients with an advanced stage of liver disease risk of hepatic decompensation during antiviral treatment was estimated as to high. Thrombocytopenia had a significant impact on the negative evaluation in 12 subjects. In addition, advanced age (> 70 years) linked to a limited physical capacity was a reason that prevented treatment in eight patients. Six individuals reported poor tolerability of the previous treatment. Two patients were pregnant at the time of presentation.(ii) In seven (6.8%) patients mainly with advanced fibrosis, a history of treatment failures and further negative predictors, the chance to achieve SVR was considered to be too low to reach an acceptable risk/benefit ratio.
- Thirty-one (30%) patients were considered to have no urgent treatment indication in our view, as the stage of liver fibrosis was not advanced limiting the benefit of an immediate treatment.
- Finally, nonmedical patient related reasons played an important role in thirty-two patients (31%). All patients were widely informed about their liver related prognosis, risks, benefits and conditions of current triple therapy concepts as well as the chances for alternative treatment options that may be accessible in the future. Eighteen patients decided to wait for future treatment options. Twelve patients were either completely lost to follow up or missed appointments and were considered as incompliant. In seven cases a triple therapy concept was not possible due to social or work related reasons i.e. two subjects were professional drivers. In Table 2 we have listed the factors that influenced treatment decision.

**Table 2:** Factors that influenced the decision not to treat with P/R/PI.

Factor			Frequency n (% of 86 patients)
**Treatment associated safety concerns**			**66 (64%)** *
	Comorbidities		48 (47%)
		*Autoimmune exacerbation*	*18 (17%)*
		*Neuro-psychiatric diseases*	*15 (15%)*
		*Cardiovascular diseases*	*8 (7.8%)*
		*Anemia*	*7 (6.8%)*
		*Other comorbidities*	*9 (8.7%)*
	Risk of hepatic decompensation		10 (9.7%)
	Thrombocytopenia¶		12 (12%)
	Age		8 (7.8%)
	Intolerance to previous P/R treatment		6 (5.8%)
	Need for other urgent procedures		6 (5.8%)
	Pregnancy		2 (1.9%)
**Poor chance for SVR**			**7 (6.8%)** *
**Treatment urgency** §			**31 (30%)** *
**Nonmedical patient related reasons**			**32 (31%)** *
	Patient wish		18 (17%)
	Poor compliance/LTFU		12 (12%)
	Social reasons (i.e. bus driver)		7 (6.8%)

* More than factor could have influenced treatment decision.

¶ Eleven patients with platelets <60/nl; one patient with a platelet count of 89/nl and several other risk factors.

§ Based on individual risk for disease progression and current stage of liver fibrosis (majority F0/F1∶71%; remaining patients with Fibroscan result <9 kPa and one patient with F2 in liver biopsy)

### Safety and Effort of Triple Therapy

Overall, 406 visits during 1022 treatment weeks (one visit every 2.5 weeks) were documented. During the investigated time period several cases of cytopenia occurred that required dose modifications of the antiviral therapy. Thirty-two patients (37%) experienced at least one episode of significant anemia (Hb<10 g/dl). In 12 (14%) of these patients, hemoglobin level dropped to a concentration of less than 8.5 g/dl. In 12 patients anemia was countered by blood transfusions. Less commonly thrombocytopenia (20%) and neutropenia (12%) reached a stage where dose modifications are recommended. Ribavirin dose reduction was required in 31 patients (36%) predominantly due to anemia. In 11 patients (13%) a temporary discontinuation became necessary. The dosage of pegylated interferon alfa was reduced in 20 (23%) patients and six (7%) required a temporary discontinuation.

Twenty-one hospitalizations related to antiviral therapy have been documented in 16 patients (19%). Most frequent reason was a severe or symptomatic anemia (62%), followed by infections (14%) and hepatic decompensations (14%). Overall, the rate of treatment-associated hospitalization was estimated as 0.99/patient treatment year. The 16 patients who were referred to hospital were at a similar age as the remaining patients (53.9 vs. 53.4 years) but more likely to have a more advanced liver disease at baseline indicated by a significantly higher MELD-Score (9.6 vs. 7.3) and a lower platelet count (107.5/nl vs. 169.9/nl). Platelets<110/nl (48% hospitalized patients) and>five points in the Child-Pugh Score (45% hospitalized patients) were associated with a high risk of hospitalization. In contrast, only four out of 60 patients (6.7%) who had none of the above-mentioned risk factors required hospitalization (Table 3).

**Table 3:** Baseline characteristics of patients who were hospitalized until week 12.

		No hospitalization until week 12	Hospitalization until week 12	p-value
Number of patients		70 (81%)	16 (19%)	
Treatment Regimen				
	P/R/TLV	43 (61%)*	12 (75%)	0.51
	P/R/BOC	19 (27%)*	2 (13%)	0.29
	P/R Lead-In¶	9 (13%)	2 (13%)	0.99
Mean Age ± SD (years)		53.4 ± 10.2	53.9 ± 11.4	0.45
Gender				
	Male (m)	49 (70%)	6 (38%)	0.14
	Female (f)	21 (30%)	10 (63%)	0.05
Treatment experienced				
	Yes	51 (73%)	12 (75%)	0.93
	No	19 (27%)	4 (25%)	0.88
Platelet Count (/nl)				
	*Mean ± SD*	170 ± 65.7	108 ± 57.3	< 0.01
	< 110	11 (16%)	10 (63%)	< 0.001
	N/A	1 (1.4%)	0 (0%)	
Hemoglobin (g/dl)				
	*Mean ± SD*	14.8 ± 1.56	13.5 ± 1.40	< 0.01
	< 13 (m),<12 (f)	3 (4.3%)	2 (13%)	0.23
ALT				
	*Mean ± SD*	100 ± 71.4	108 ± 94.7	0.39
	< ULN	9 (13%)	4 (25%)	0.26
	1–2×ULN	32 (46%)	4 (25%)	0.25
	2–5×ULN	25 (36%)	6 (38%)	0.91
	> 5×ULN	4 (5.7%)	2 (13%)	0.36
Fibrosis stage				
	F0–F2	10 (14%)	1 (6.3%)	0.41
	F3/F4	59 (84%)	15 (94%)	0.75
	N/A	1 (1.4%)	0 (0%)	
De Ritis ratio				
	≤ 1	50 (71%)	8 (50%)	0.35
	> 1	20 (29%)	8 (50%)	0.18
Child-Pugh Score				
	5	58 (83%)	9 (56%)	0.34
	> 5	6 (8.6%)	5 (31%)	< 0.05
	N/A	6 (8.6%)	2 (13%)	
MELD				
	*Mean ± SD*	7.33 ± 2.24	9.60 ± 2.89	< 0.01
	≤ 8	56 (80%)	8 (50%)	0.19
	> 8	8 (11%)	7 (44%)	< 0.01
	> 10	2 (2.9%)	5 (31%)	< 0.001
	N/A	6 (8.6%)	1 (6.3%)	

P: pegylated interferon alfa; R: ribavirin; TLV: telaprevir; BOC: boceprevir; N/A: not available; n.d.: not differentiated; SD: standard deviation

* One patient switched from TLV to BOC after week 2 of therapy

¶ Patients that discontinued treatment during/after the lead-in phase and never received a PI

### Treatment Failure at Week 12

**Figure 2:**
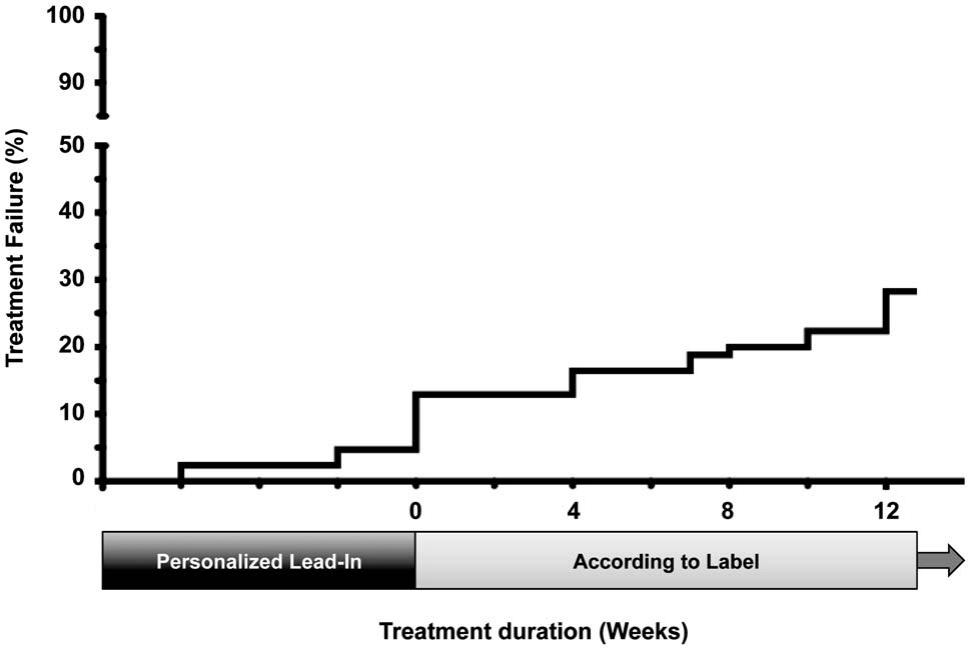
Treatment failures during the observed time period.

Of the 86 patients that were started on a triple therapy concept 20 (23%) dropped out before week 12 of the approved treatment regimen. In 10 of these patients treatment was stopped due to a virological failure, while seven had to discontinue because of AEs and one patient died after a gastrointestinal infection. In two patients, both poor tolerability as well as poor virological response contributed equally. Of those, who maintained on therapy, four patients had to stop at week 12 because they met futility criteria. In addition, one patient experienced a SAE at week 12 of therapy that resulted in a permanent discontinuation. As a result, 25 out of 86 patients (29%) had to be classified as a treatment failure at week 12 (Figure 2).

Patients that discontinued had a significantly lower platelet count at baseline (123/nl vs. 172/nl, p<0.001). More than half of the patients with a baseline platelet count of less than 110/nl had to be classified as a treatment failure at week 12 of therapy. Patients infected with HCV genotype 1a were more likely to experience a treatment failure (p<0.05). IL28B CC genotype was associated with a superior treatment outcome. None of the 10 patients with the IL28B CC genotype that started a triple therapy experienced a virological failure until week 12 of treatment. However, one patient experienced a lethal AE. No significant difference could be observed between TLV and BOC in those receiving at least one dose of PI (p = 0.73) (Table 4).

**Table 4:** Baseline characteristics of patients with and without treatment failure until week 12.

		Continued treatmentafter week 12	Treatment failureuntil week 12	p-value
**Number of patients**		61 (71%)	25 (29%)	
**Mean Age ± SD (years)**		52.8 ± 11.2	55.4 ± 7.81	0.12
**Gender**				
	Male (m)	38 (62%)	17 (68%)	0.76
	Female (f)	23 (38%)	8 (32%)	0.69
**HCV genotype**				
	1a	13 (21%)	13 (52%)	< 0.05
	1b	47 (77%)	11 (44%)	0.13
	Mixed/n.d.	1 (1.6%)	1 (4%)	0.49
**IL28B**				
	CC	9 (15%)	1 (4%)	0.16
	Non-CC	43 (70%)	22 (88%)	0.06
	N/A	9 (15%)	2 (8%)	
**Treatment experienced**				
	Yes	47 (77%)	16 (64%)	0.52
	No	14 (23%)	9 (36%)	0.29
**Platelet count (/nl)**				
	*Mean ± SD*	172 ± 69.3	123 ± 52.5	< 0.001
	<110	10 (16%)	11 (44%)	< 0.05
**Hemoglobin (g/dl)**				
	*Mean ± SD*	14.7 ± 1.66	14.4 ± 1.47	0.24
	< 13 (m),<12 (f)	3 (4.9%)	2 (8%)	0.56
**ALT**				
	*Mean ± SD*	96.3 ± 70.4	115 ± 87.8	0.17
	< ULN	11 (18%)	2 (8%)	0.28
	1–2×ULN	24 (39%)	12 (48%)	0.57
	2–5×ULN	22 (36%)	9 (36%)	1
	> 5×ULN	4 (6.6%)	2 (8%)	0.82
**Fibrosis stage**				
	F0–F2	8 (13%)	3 (12%)	0.88
	F3/F4	52 (85%)	22 (88%)	0.95
	N/A	1 (1.6%)	0 (0%)	
**De Ritis ratio**				
	≤ 1	42 (69%)	16 (64%)	0.80
	> 1	19 (31%)	9 (36%)	0.29
**Child-Pugh Score**				
	5	48 (79%)	19 (76%)	0.67
	> 5	6 (9.8%)	5 (20%)	0.29
	N/A	7 (11%)	1 (4%)	

N/A: not available; n.d.: not differentiated; SD: standard deviation.

**Figure 3:**
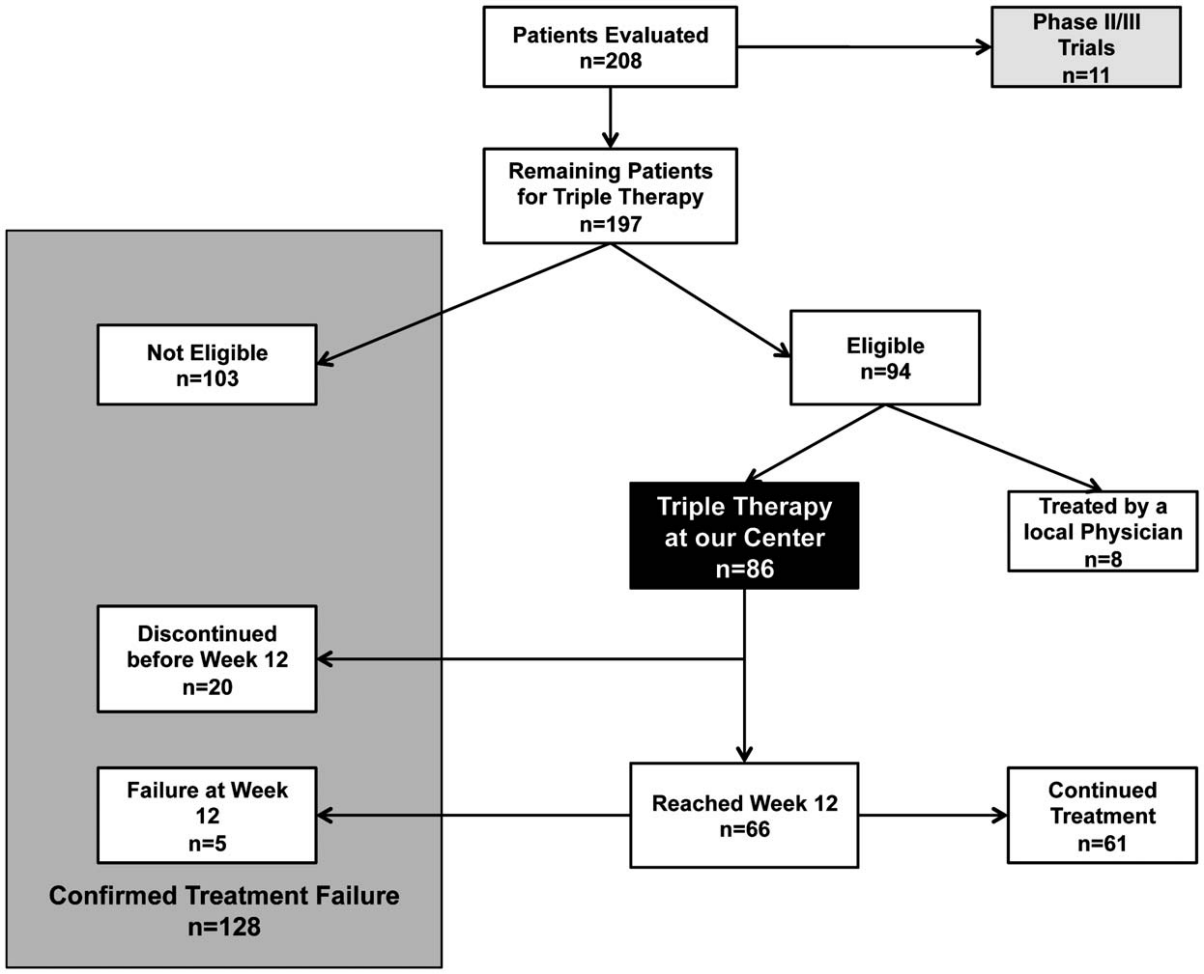
Overall outcome of the evaluation process and treatment period.

Overall, 128 patients (65%) were either not eligible or experienced a treatment failure at week 12 (Figure 3).

## Discussion

Pivotal phase III trials investigating the efficacy and safety of triple therapy including boceprevir or telaprevir showed excellent response rates with a reasonable safety profile. However, considerable concerns have been raised to what extend the new treatment concepts can be translated into clinical practice as only highly selected patients qualified for registration trials [11]. At this stage it remains unclear what proportion of the total HCV genotype 1-infected population is eligible for treatment and which patients will subsequently benefit from the novel therapeutic options. Here we describe our initial experience in selecting patients for and treating with PI-based antiviral treatment regimens. Selection of patients for treatment was based on four main factors, which were treatment-associated safety concerns, chance for SVR, treatment urgency and nonmedical patient related reasons. It has to be considered that the here studied patient population has already been preselected by the referral approach. Patients with decompensated cirrhosis and other obvious contraindications for therapy are managed by other clinics at our center, i.e. by the liver transplant unit (Figure 1). Still, so-called difficult-to-treat patients including those with advanced liver disease, previous treatment failures and individuals with comorbidities were overrepresented in this cohort. Subsequently, safety concerns for P/R/PI played a major role for not selecting patients for therapy. On the other side, mild liver disease and patient’s wish were also frequent reasons for not initiating therapy at this stage with more convenient interferon free regimens on the horizon (11, 13). Interestingly, poor chance to achieve SVR played a minor role not to start therapy mainly due to high expectations in efficacy and the opportunity of the lead-in phase. In the end, treatment was not initiated in half of the patients, including several patients with the most urgent medical need as well as individuals likely being the easiest to treat. Considering the referral approach to our hepatitis treatment unit, we suggest that the proportion of HCV-infected patients not qualifying for current treatment options can be estimated to be even higher than 50% if the here used evaluation criteria are applied.

Management of adverse events was an enormous effort in this cohort. Patients were seen almost every two weeks at our outpatient clinic and the overall frequency of consultations was certainly considerably higher since we did not assess visits at the general practitioner, local hospitals and telephone calls. Although we only analyzed the first treatment period, hospitalization became already necessary in nearly one out of five patients. Anemia was the most prevalent side effect requiring frequent hemoglobin monitoring and blood transfusions.

Infections represent the most serious complication of interferon alfa-based treatment of hepatitis C. Death of one patient during the first 12-week treatment period and an additional death of a patient at week 14 was related to gastrointestinal infection with sepsis.

In contrast to the registration trials a much larger proportion of patients had to stop therapy early during therapy. Both virological treatment failure and adverse events accounted for these early treatment discontinuations. An obvious explanation for virological failure might be the large number of patients with F3/F4 fibrosis and previous treatment failure [19]. In addition, advanced liver fibrosis has been shown to be linked to a higher incidence of adverse events [12]. In our cohort HCV genotype 1a infection and low platelet counts were associated with early treatment failure. The impact of platelet counts suggests that the negative predictive value of liver cirrhosis increases with more advanced stages of cirrhosis.

Platelet counts also predicted the need for hospitalizations during therapy further highlighting the value of this specific marker in the context of new antiviral therapies. Of note only six out of 14 patients with platelet counts of less than 90/nl, which is the recommended cut-off level for treatment eligibility with P/R [20], managed to pass week 12 futility rules. Our data indicate that even higher platelet levels have to be considered as a predictive marker for poor treatment outcome since six out of seven patients with a baseline platelet count between 90 and 110/nl either needed to be referred to hospital and/or experienced a virological failure. Platelets<110/nl were also significantly associated with treatment failure at week 12. By further follow-up, 70% of patients with platelets<110/nl required hospitalization at some point and 2 of these patients died (data not shown). We therefore suggest that low platelet count of<110/nl is a marker for advanced liver disease with a high risk for serious adverse events during P/R/PI treatment. In addition, more than 5 points in the Child-Pugh score was another valuable predictive marker for adverse events and hospitalization.

Our study was not designed to directly compare the two available PIs. Many of the observed adverse events are certainly related to P/R since some patients discontinued treatment even before taking a single dose of a PI. It was not the aim of this study to attribute treatment-associated risk and effort to a single component of the antiviral therapy regimen. We could already demonstrate that, in contrast to pivotal registration trials, safety and efficacy of currently available antiviral regimens are limited in a real world cohort. In addition treatment required enormous recourses both in terms of time and monitoring visits as well as by the management of side effects. On the other side HCV RNA became undetectable in most of the patients who reached week 12 of therapy. Thus it is crucial to identify reliable markers for the prediction of both safety and efficacy. In our opinion a lead-in phase regardless of the later used PI can be a valuable tool in patients with uncertain treatment tolerability and offer additional information on chances for SVR. Early discontinuation may prevent SAEs and even some lethal complications. According to our data platelet count and Child-Pugh Score, markers for advanced liver disease, seem to be valuable tools to identify patients with a high therapy associated risk and a poor treatment outcome. However, specific cut-offs to determine ineligibility for triple therapy (i.e. platelet count <110/nl, Child-Pugh Score >5) warrants further validation in larger cohorts. Still, the risk/benefit ratio should be well calculated in patients with advanced cirrhosis indicated by such risk factors. If treatment will be initiated in such patients a very close monitoring and early management of adverse events is essential.

We here presented our first experiences with new triple therapies, rising considerable safety concerns at least in certain populations. However, overall safety of these new treatments will certainly improve with more “real world” data and more experience gained regarding the optimal management of adverse events in particular anemia. According to recently published data RBV dose reduction as first line strategy is done much more rapidly in our center, which seemed to decrease the number of hospitalizations in following cohorts [21]. More effective strategies to meet severe infections need to be developed. High efficacy of PI-based therapies raises high ambitions to treat a huge amount of patients. This may lead to an underestimation of risk factors. Our data suggest, that patients need to be selected very carefully since a sensible patient selection is the first and may be the most important step to ensure a reasonable safety profile and a high efficacy. From a retrospective point of view we might slightly shift our patient selection to a cohort with less advanced liver disease. In our opinion ideal candidates for current PI based therapies are those with middle stage fibrosis (F2–F3) and well-compensated cirrhosis as well as prevalence of some positive predictors for SVR. Including some more easier-to-treat patients would certainly reduce the huge effort that is required for therapy management. A more balanced patient cohort may also permit to treat a higher number of HCV-infected individuals even more cost-effectively. Conclusions of our study are supported by a recently published, huge epidemiologic study comparing different approaches to the initiation of antiviral treatment. Here it has been demonstrated that starting treatment as soon as liver fibrosis has reached F2 might be a more effective strategy than delaying treatment until higher stages of fibrosis have been established [22]. We here identified valuable pretreatment markers to predict both safety and efficacy, which may help to select the appropriate patients in the future. However, further studies will certainly be necessary to develop a valuable scoring system for this selection process. According to the poor outcome of patients with advanced disease and the small benefit for those at very early stages of the disease, it has to be concluded that despite the improvements that have been achieved during the last year safer and more efficient treatment options are still urgently needed.
